# Phytochemicals in Human Milk and Their Potential Antioxidative Protection

**DOI:** 10.3390/antiox7020032

**Published:** 2018-02-22

**Authors:** Apollinaire Tsopmo

**Affiliations:** 1 Food Science and Nutrition Program, Department of Chemistry, Carleton University, 1125 Colonel By Drive, Ottawa, ON K1S 5B6, Canada; apollinaire_tsopmo@carleton.ca; Tel.: +1-613-520-2600 (ext. 3122); 2 Institute of Biochemistry, Carleton University, 1125 Colonel By Drive, Ottawa, ON K1S 5B6, Canada

**Keywords:** oxidative stress, human milk, infant, phytochemicals

## Abstract

Diets contain secondary plant metabolites commonly referred to as phytochemicals. Many of them are believed to impact human health through various mechanisms, including protection against oxidative stress and inflammation, and decreased risks of developing chronic diseases. For mothers and other people, phytochemical intake occurs through the consumption of foods such as fruits, vegetables, and grains. Research has shown that some these phytochemicals are present in the mother’s milk and can contribute to its oxidative stability. For infants, human milk (HM) represents the primary and preferred source of nutrition because it is a complete food. Studies have reported that the benefit provided by HM goes beyond basic nutrition. It can, for example, reduce oxidative stress in infants, thereby reducing the risk of lung and intestinal diseases in infants. This paper summarizes the phytochemicals present in HM and their potential contribution to infant health.

## 1. Introduction

Plant secondary metabolites, often referred to as phytochemicals, are believed to play an important role in human health. Benefits include the protection against oxidative stress, inflammation; and reduction in risks factors of chronic conditions, such as heart diseases, cancer, diabetes, and neurodegenerative disorders [1,2]. Oxidative stress is present in all of these ailments and antioxidant phytochemicals have been widely investigated in the adult population for their roles in quenching or reducing excess oxidants, thereby restoring the redox balance. For newborns, human milk (HM) represents the primary and preferred source of nutrition and there are data in the literature showing that the benefits of HM go beyond basic nutrition [3].

Human milk from well-nourished mothers is believed to meet the nutrient requirements of infants for up to six months because its composition is dynamic and varies with the mother’s diet and time postpartum. The dynamic changes in the composition of HM with time of lactation is to match the changing needs of growing infants. Proteins in HM are sources of nitrogen, amino acids and peptides for the newborn. Proteins, specifically those from the whey fraction are also involved in the development of the immune system, while lactoferrin from the casein group contributes to non-immunologic defence [4]. HM proteins can also serve as a source of antioxidant peptides [5,6]. As well, glutamate, present in HM, can act as a major oxidative fuel for enterocytes and promote gastrointestinal barrier function [7]. Oligosaccharides and polysaccharides in HM can inhibit the adhesion of bacteria to the surface of epithelial cells or promote the development of bifidus flora, thereby contributing to the prevention of infectious diseases in the newborn [8,9]. Oligosaccharides can also decrease the likelihood of injury to the retina and the lung in premature infants with respiratory distress syndrome [10,11]. HM lipids contain a considerable amount of long chain polyunsaturated fatty acids, which are precursors of prostaglandin-like prostacyclins that can improve ventricular function in infants [12]. These fatty acids are also essential components of membrane-rich tissues, such as the brain and the retina photoreceptor membrane [13]. HM provides bioactive agents that include antimicrobial (e.g., immuloglobulins), anti-inflammatory (e.g., lactoferrin) and bioactive peptides. In addition, there are data demonstrating that breastfeeding promotes the development of the infant immune system and this might confer long-term health outcomes [14]. However, the benefit of HM goes beyond that of proteins, oligosaccharides and lipids because phytochemicals from mothers’ diets are transferred to their milk. Several of the phytochemicals in HM have antioxidant activities that may help the infant cope with oxidative stress. The aim of this review is to describe antioxidant phytochemicals present in the mother’s milk and their potential contribution to redox balance in infants.

## 2. Phytochemicals in Human Milk

Polyphenols are one of the largest groups of phytochemicals present in crops. Thousands of phenolic structures have been identified, of which about half belong to the class of flavonoids. This class is further sub-divided into flavones, isoflavones, flavanones, catechins and anthocyanins. Polyphenols have been studies in various systems (in vitro and in vivo) and they possess biological activities, such as anti-inflammatory and antioxidant activities. In addition, they can regulate the activity of many enzymes [15,16]. These activities are associated with the promotion of vascular health, cognitive function, redox balance, hormonal balance, or neuronal function [15,17]. One of the most common biological functions of polyphenols is their ability to act as antioxidants, thereby potentially protecting adults against oxidative stress and inflammation, while, at the same time, decreasing the risk of developing chronic and degenerative diseases (e.g., macular degeneration, cancer, obesity, diabetes) [18,19]. Oxidative stress is also present in infants and is associated with respiratory and intestinal diseases [20,21].

### 2.1. Flavonoids in Human Milk

**Figure 1:**
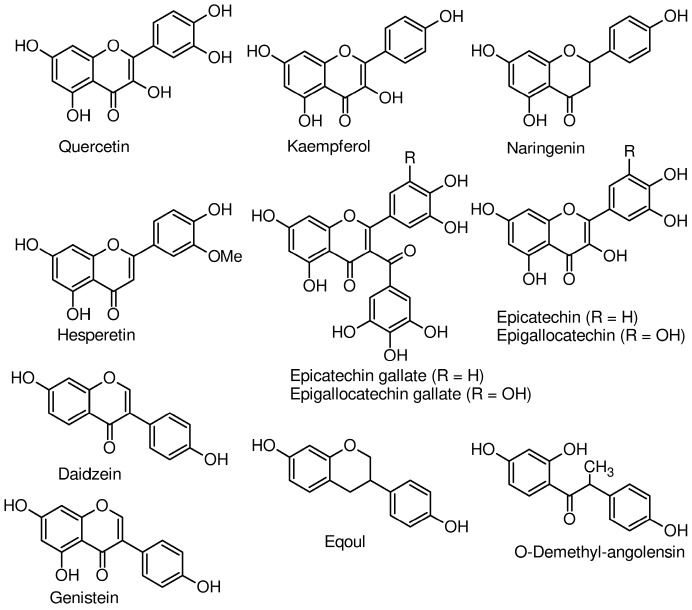
Chemical structures of polyphenols detected in human milk.

Secondary metabolites are classified into classes including polyphenols, of which flavonoids constitute the largest sub-group. Structures of some flavonoids identified in the mother’s milk are presented in Figure 1 and their concentrations in Table 1. A study conducted by Song et al. [18] detected seven flavonoids—epicatechin, epicatechin gallate, epigallocatechin gallate, naringenin, kaempferol, hesperetin, and quercetin—in milk of mothers who gave birth to full term babies. Mean concentrations at one week postpartum varied from 15.7 nmol/L for kaempferol to 1118.8 nmol/L for epigallocatechin gallate. An ingestion of roasted soybeans (20 g, equivalent to 37 mg isoflavones) resulted in mean total isoflavone concentrations of about 0.2 μmol/L in breast milk, with the main constituents being daidzein and genistein [22]. In the work of Khymenets et al. [23], the consumption of dark chocolate led to the identification of epicatechin and its metabolites 12 h after ingestion in the HM of mothers obtained at 6 months postpartum. The metabolites were sulfates and glucuronates of epicatechin, metoxy-catechin, and γ-valerolactone [23]. In nursing mothers who consumed a soy beverage containing 55 mg of total isoflavones for 2–4 days, isoflavone contents of their milk increased from 5.1 to 70.7 nmol/L, while amounts in the urine of their infants went from 29.8 to 111.6 nmol/mg creatinine [24]. In addition, the mean isoflavone concentration in the plasma of these infants was 19.7 nmol/L. Data from this research is an indication that isoflavones are available in infants and can potentially protect them from oxidative stress because they are known antioxidant molecules. In a related study, nursing women who received 250 mL of soy drink with an isoflavone content of 12 mg for 6 days had 12 nmol of isoflavone/L in their milks [25]. Compared to the study of Franke et al. [24], 12 nmol of isoflavone/L of HM seems small but this is because the two soy drinks had different amounts of isoflavone (12 mg vs. 55 mg). In another study, breastfeeding women received meals that provided 1 mg of quercetin/kg bodyweight. In milks collected after 12 h, its mean concentration was 68 ± 8 nmol/L and represented about a 1.7-fold increase relative to values before and at 48 h after the supplementation [26].

### 2.2. Carotenoids

**Figure 2:**
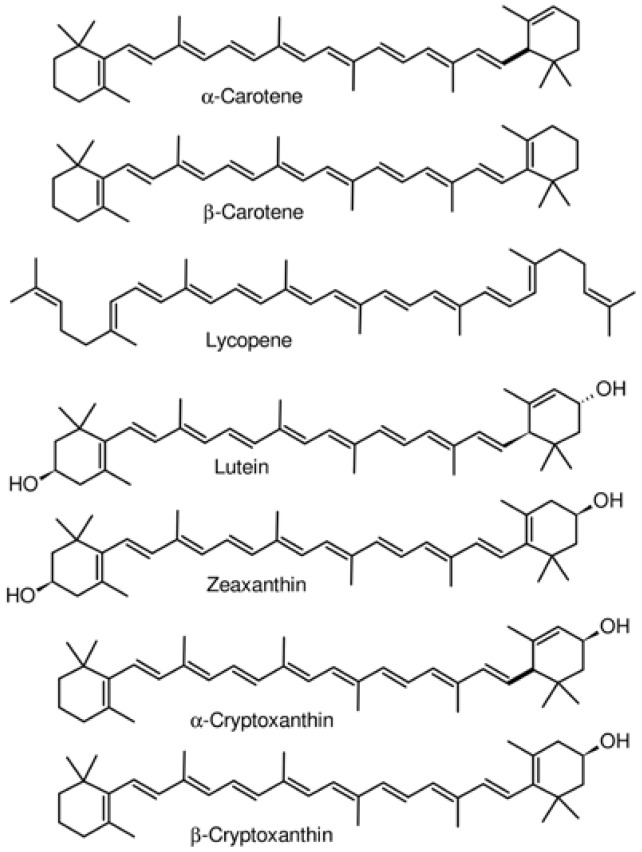
Chemical structures of carotenoids detected in human milk.

The other abundant class of phytochemicals in human milk is the carotenoids (Figure 2, Table 1). Dietary supplementation of lactating mothers with antioxidant rich foods has an influence on how much is present in milk and therefore, the exposure of infants to these molecules. In the study conducted by Haftel et al. [27], women took 15 mg β-carotene or 15 mg of lycopene, in the form of carrot puree or mashed tomato, per day. The two carotenoid molecules were detected in HM and their concentrations increased with time to reach maximum values after two or four days, depending on the individual. Lycopene levels rose to a maximum of 130%, and β-carotene to a maximum of 200%, relative to baseline values [27]. In a related work, seven carotenoids were detected in HM collected one to thirteen weeks postpartum from free living mothers (i.e., no diet intervention). Amongst them, β-carotene (164.3–88.0 nmol/L), lutein (121.2–56.4 nmol/L) and lycopene (119.9–49.5 nmol/L) were the most abundant [18]. The concentrations of others were α-cryptoxanthin (30.6–13.5 nmol/L), β-cryptoxanthin (57.4–24.8 nmol/L), and zeaxanthin (46.3–21.4 nmol/L). The amount of each carotenoid decreased from week 1 to week 13 [18]. The variation in the concentration of each of the carotenoid molecules was most likely due to the oxidative status of the mother or to the amount in their diet, although the study did not collect information on the participants’ diets. In another study, pregnant women received daily, 6 g of Chlorella, a single-cell green algae rich in carotenoids, from 16–20 weeks of gestation until the day of delivery [28]. There were significant increases of 1.7, 2.6 and 2.7-fold in β-carotene, lutein and zeaxanthin, respectively in HM of the experimental group, compared to the control group, at 0–6 days postpartum. A recent study quantified carotenoids in donors’ and lactating mothers’ milk and found that concentrations of α-carotene, β-carotene, lycopene and β-cryptoxanthin were 1.9 to 5.7-fold lower in the donors’ milk samples [29]. Lower contents of carotenoids in donor milk could be due to the pasteurization of milk necessary to prevent microbial growth and ensure its safety [30], but storage might contribute to the reduction as well. Donor milk is an effective alternative source of nutrition, specifically for preterm infants, when the mother’s own milk is not available. Information on whether the amount of antioxidant phytochemicals present in donors’ milk has an effect on oxidative stress related outcomes in the preterm infant is not available. Phytochemicals (e.g., flavonoids and carotenoids) have antioxidant properties and their presence in diets might protect pregnant women and their fetuses against oxidative stress induced during pregnancy. The protection can continue after birth because some of the phytochemicals have been detected not only in HM, but also in biological fluids (e.g., blood and urine) [31]. In fact, there are direct correlations between concentrations of HM lutein with its daily intake and this has led to the recommendation by some institutions to increase fruit and vegetable intakes throughout the duration of pregnancy and lactation [32].

### 2.3. Other Phytochemicals

Three garlic acid metabolites, known as allyl methyl sulfide, allyl methyl sulfoxide and allyl methyl sulfone, were detected in breast milk 2.5 h after the consumption of garlic [36]. Allyl methyl sulfide affected the odor of milk but showed antioxidant activity, characterized by its ability to reduce the rate of oxidation of cumene [37]. Both odor and antioxidant characteristics of allyl methyl sulfide are due to the presence of sulfur. Caffeine and its catabolic products, theobromine, and xanthine, are key molecules in tea and coffee. Their concentrations and those of related molecules, theophylline and paraxanthine, in HM were determined to vary from 0.06 to 0.77 µg/mL [38]. Caffeine, theobromine and xanthine have been found in model systems to quench hydroxyl radicals, thereby preventing oxidative DNA breakage induced by this radical species [39]. Meanwhile, the effects of caffeine and its congeners at concentrations detected in HM on the biochemistry of HM or on newborn outcomes are unknown.

## 3. Oxidative Stress in Infants

The higher production of oxygen-derived metabolites, collectively known as reactive oxygen species (ROS) in aerobic organisms, compared to the concentration of available antioxidant molecules and enzymes is termed oxidative stress. The presence of excess ROS is an important mediator of cell and tissue damage [20,40]. Biological molecules susceptible to oxidation include lipids, proteins, and nucleotides [41,42]. In general, organisms prevent oxidative damage by maintaining a critical oxidation–reduction balance, but this is not always the case in the presence of diseases, external stimuli, improper nutrition or exposure to a hyperoxic environment, as encountered at birth. Data exist to show that the transition from an intrauterine to an extrauterine life is characterized by physiological and metabolic changes due, in part, to an increase in the availability of oxygen and a high level of free iron that can enhance the production of highly toxic hydroxyl radicals through the Fenton reaction [40]. Newborns, specifically those who are premature, cannot efficiently deal with oxygen at relatively high concentrations compared with the intrauterine environment because antioxidant enzymes mature during late stage gestation and also, because of inadequate transfer of antioxidants, like vitamins E, C, β-carotene, and ubiquinone, across the placenta [43]. 

The evaluation of oxidative stress in newborns is based on the quantification of antioxidant molecules, enzyme activities or markers of lipids and proteins, or DNA damage. For example, malondialdehyde (MDA), a marker of lipid peroxidation and 8-hydroxy-2′-deoxyguanosine, a maker of DNA damage, is higher in the cord blood of preterm low birth weight infants [44]. Higher concentrations of protein carbonyls were reported in neonatal lungs of subjects with bronchopulmonary dysplasia [45], while in infants treated with supplemental oxygen, ortho-tyrosine, a marker of protein oxidation, increased with increasing inspired oxygen [46]. Perinatal hypoxia increased the oxidation of lipids in cord blood and also decreased the concentration of the intracellular antioxidant peptide, glutathione [47]. Oxidative stress in newborns has been linked to several conditions. Some of these are chronic lung diseases or bronchopulmonary dysplasia, a condition that usually occurs in preterm infants receiving respiratory support with mechanical ventilation or prolonged oxygen supplementation [48]. Other oxidative stress-associated conditions are necrotizing enterocolitis, an inflammation of the small intestine and bowel surface, with infiltration of epithelial cells by bacteria; and retinopathy of prematurity, a type of oxygen-induced damage to blood vessels in the retina that are undergoing neovascularization [49,50].

There are several strategies for reducing oxidative stress in newborns including supplementation with enzymatic or non-enzymatic antioxidants [51]. Meanwhile, human milk seems to provide better antioxidant protection in early life due, in part, to its ability to scavenge free radicals compared to formulas [52]. This might be due to the presence of the antioxidant enzymes—glutathione peroxidase, catalase, and superoxide dismutase—present in HM but not in formula [53], which, in addition to their antioxidant effects in the gut, may pass through the porous neonatal intestine early in infancy [52]. In addition to enzymes, vitamins E and C, and possibly phytochemicals can contribute to the protection provided by HM.

## 4. Antioxidant Phytochemicals in Human Milk and Redox Balance in Infants

Secondary plant metabolites, and specifically those with antioxidant and anti-inflammatory properties, play an important role in human health. Human milk (HM) is the optimal food for newborns and is, in many cases, the only source of nutrition for up to six months. The presence of plant antioxidant molecules in HM, like polyphenols and carotenoids, indicates that they might have a role in newborn health outcomes. There are several reviews on the contribution of polyphenols in the management of oxidative stress and related conditions in the adult population [17,54] but not in infants. The effect of the consumption of dietary polyphenols through HM on the health of infants is not entirely understood because only a few studies have attempted to determine the availability of polyphenols in HM of lactating mothers and their potential accessibility to HM-fed infants [23]. The effect can be studied by analyzing phytochemicals in HM and how they affect milk stability or by quantifying the amount of these molecules in infant bio-fluids and relating this to health outcomes in which oxidative stress plays a role. The total concentration of polyphenols in HM, collected three days after parturition, inversely correlated with malondialdehyde, a genotoxic product of lipid peroxidation, indicating an increase stability of milk from mothers with high intake of vegetables that are rich in antioxidant phytochemicals [55]. A recent study found that the carotenoid content of HM samples decreased with an increasing lactation period but, for flavonoids, there was only minimal or, in certain cases, no change in content with the stage of lactation [18]. How this affects the oxidative stability of HM is unknown because it was not part of that study. Other works have been conducted to determine the antioxidant potential of HM collected at various stages of lactation and the information was recently reviewed [56]. Although, in one of the studies, total antioxidant capacity of HM was correlated with α-tocopherol concentration [57], none of the studies looked at the oxidative stability of milk with regard to the content of their antioxidant phytochemicals.

Carotenoids are known for their antioxidant properties and this can enhance the immune system and visual acuity because of their accumulation in the eye. The deposition of lutein and zeaxanthin, for example, in the human retina occurs early in life [58], and their content in HM may then be critical to the development of the infant visual acuity. The macular pigment optical density in the retina of healthy full term infants significantly correlated with concentrations of zeaxanthin in their serum samples (*r* = 0.68) and in their mother’s serum (*r* = 0.59) [58]. Additionally, the same work reported mother–infant correlations for total serum carotenoids and skin carotenoids, indicating further potential contribution of this group of phytochemicals to infant development. The retina is exposed to an intense energy source from lens focused light that generates free radicals [59]; the presence of carotenoids in the eye can consequently improve infant visual acuity while also preventing oxidative stress. Lactating mothers with low intakes of carotenoids might possibly expose their infants to less protection from oxidative stress. Fruits and vegetables are recommended throughout the duration of pregnancy and lactation to maintain sufficient amounts of carotenoids [28] and possibly, to better protect infants. In a study by Perrone et al. [60], newborns received lutein at 12 h and 36 h after birth. The quantification of hydroperoxides, a maker of lipid oxidation in the cord blood, at 48 h of life in infants, showed a significant reduction in oxidative stress in the lutein group compared to the control group [60]. This is an indication of a decrease oxidation of lipids in infants due to the antioxidant nature of lutein. In a related work, a combination of lycopene, lutein, and β-carotene given to preterm infants decreased C-reactive protein in plasma and improved rod photoreceptor sensitivity [61]. A possible mechanism for this could be through an antioxidant mechanism that prevented oxidative damage to the photoreceptor.

The exposure of infants to the flavonoid, quercetin, through HM was estimated to be 0.01 mg/day based on the assumption that they consumed 900 mL/day of milk, equivalent to about 45 nmol quercetin/L [26,62]. In a related work, mothers who consumed 20–25 mg of isoflavones daily might have exposed their breastmilk fed infants to 0.005–0.01 mg/day of this group of polyphenols [63]. The contribution of flavonoids to the reduction of oxidative stress in infants is not clear, although genistein, daidzein and glycitein were detected in the urine of 4 to 6 month old infants fed soy products [64]. An increase of 14-fold in isoflavone content was found in the milk of lactating mothers who consumed soy products, concomitantly with an increase of 4-fold in the urine of their babies [24]. The presence of flavonoids in biological fluids of infants is an indication that they might help them cope with oxidative stress, although evidence is needed from future studies.

## 5. Conclusions

Carotenoids found in human milk may play a role in its oxidative stability and in infant redox balance, inflammatory status and visual acuity. The minimum concentrations needed to provide protective effects are not available. This is due, at least in part, to the limited number of studies that have correlated carotenoid contents in human milk to a specific infant health outcome. The contribution of flavonoids, the other main group of antioxidant phytochemicals in human milk, to infant oxidative status is even less clear. Despite this, the recommendation to consume more fruits and vegetables during both pregnancy and lactation is a key component of dietary guidelines to boost phytochemicals and protect mothers and infants from oxidative damage and related diseases.

## Figures and Tables

**Table 1:** Concentrations of phytochemicals found in human milk.

Compound	Concentration (nmol/L)	Information on Mothers and Milk
Epicatechin	63.7–828.5	Free living mothers, milk at 1, 4 and 13 week [18]
Epicatechin gallate	55.7–645.6	
Epigallocatechin gallate	215.1–2364.7	
Naringenin	64.1–722.0	
Kaempferol	7.8–71.4	
Hesperetin	74.8–1603.1	
Quercetin	32.5–108.6	Free living mothers, milk at 1, 4 and 13 week [18]
	68 ± 8.44	Diet with 1 mg quercetin/kg of body weight [26]
Lutein	56.4–121.2	Free living mothers, milk analyzed at 1, 4 and 13 weeks [18]
	497–824	Chlorella supplementation, 6 months from gestational week 16–20 until delivery [28]
	280 ± 22	Free living mothers. Milk collected at day 3 [32]
Zeaxanthin	46.3–21.4	Free living mothers, milk at 1, 4 and 13 weeks [18]
	33.2±17.2	Healthy women. Milk collected at days 2–6 [33]
α-Cryptoxanthin	13.5–30.6	Free living mothers, milk at 1, 4 and 13 weeks [18]
β-Cryptoxanthin	24.8–57.4	Free living mothers, milk at 1–14 weeks [18,34]
α-Carotene	23.2–59.0	Free living mothers, milk at 1, 4 and 13 weeks [18]
β-Carotene	88.0–164.3	Free living mothers, milk at 1–14 weeks [18,34]
	75–400	Supplementation, 30 mg β-carotene/d for 28 days [35]
	275–484	Chlorella supplementation 6 months, from gestational week 16–20 until day of delivery [28]
Lycopene	119.9–49.5	Free living mothers, milk analyzed at 1, 4 and 13 weeks [18,34]
	86–244	Chlorella supplementation for 6 months, from gestational week 16–20 until day of delivery. Milk collected at 1–6 days [28]
Isoflavones	70.7 ± 19.2	Soy beverage with 55 mg isoflavones daily for 2–4 days [24]
	12.0	Soy drink, 12 mg isoflavones daily for 6 days [25]
Epicat-Gluc-4 *	0.0–36.4	Free living mothers, milk collected at 1–30 days [23]
Epicat-Sulf-3 *	0.0–14.5	
MetEpicat-Sulf-3 *	0.0–23.7	
Caffeine **	0.06–0.77	Milk of habitual coffee and chocolate mothers [34]
Theobromine	0.08–0.50 **	
Paraxanthine	0.15–1.68 **	
Theophylline	0.10–0.66 **	

* Epicatechin metabolites; ** Concentrations expressed as µg/mL.
